# Mesenchymal hamartoma of the liver in a young adult female: a diagnostic challenge mimicking hepatic adenoma

**DOI:** 10.1093/jscr/rjag474

**Published:** 2026-06-15

**Authors:** Yuya Saruta, Yoshikuni Kawaguchi, Yujiro Nishioka, Akihiko Ichida, Takeshi Takamoto, Nobuhisa Akamatsu, Kiyoshi Hasegawa

**Affiliations:** Hepato-Biliary-Pancreatic Surgery Division, Department of Surgery, Graduate School of Medicine, The University of Tokyo, 7-3-1 Hongo, Bunkyo-ku, Tokyo, 113-0033, Japan; Hepato-Biliary-Pancreatic Surgery Division, Department of Surgery, Graduate School of Medicine, The University of Tokyo, 7-3-1 Hongo, Bunkyo-ku, Tokyo, 113-0033, Japan; Hepato-Biliary-Pancreatic Surgery Division, Department of Surgery, Graduate School of Medicine, The University of Tokyo, 7-3-1 Hongo, Bunkyo-ku, Tokyo, 113-0033, Japan; Hepato-Biliary-Pancreatic Surgery Division, Department of Surgery, Graduate School of Medicine, The University of Tokyo, 7-3-1 Hongo, Bunkyo-ku, Tokyo, 113-0033, Japan; Hepato-Biliary-Pancreatic Surgery Division, Department of Surgery, Graduate School of Medicine, The University of Tokyo, 7-3-1 Hongo, Bunkyo-ku, Tokyo, 113-0033, Japan; Hepato-Biliary-Pancreatic Surgery Division, Department of Surgery, Graduate School of Medicine, The University of Tokyo, 7-3-1 Hongo, Bunkyo-ku, Tokyo, 113-0033, Japan; Hepato-Biliary-Pancreatic Surgery Division, Department of Surgery, Graduate School of Medicine, The University of Tokyo, 7-3-1 Hongo, Bunkyo-ku, Tokyo, 113-0033, Japan

**Keywords:** adult mesenchymal hamartoma of the liver, surgical resection, robot-assisted hepatectomy

## Abstract

Mesenchymal hamartoma of the liver (MHL) is a rare benign lesion usually seen in infants and young children; adult onset is exceptional and can mimic a broad spectrum of hepatic neoplasms on imaging, including hepatocellular, cholangiocytic/biliary, and mesenchymal tumors. A 31-year-old woman was found to have a 23-mm hyperechoic mass in segment 7 on screening ultrasonography. One year later, it had enlarged to 68 mm. Contrast-enhanced computed tomography showed a well-circumscribed 75-mm lesion with gradual heterogeneous enhancement. Given her >10-year history of oral contraceptive use, a benign hepatocellular tumor such as hepatocellular adenoma was suspected; however, biliary epithelial tumors and mesenchymal tumors were also considered in the differential diagnosis. Because of rapid growth and proximity to the right hepatic vein, robot-assisted extended posterior sectionectomy with right hepatic vein resection was performed. Pathology revealed fibromyxoid stroma with spindle mesenchymal cells, irregular bile ducts, consistent with MHL without malignant change.

## Introduction

Mesenchymal hamartoma of the liver (MHL) is an uncommon benign tumor-like lesion composed of varying proportions of mesenchymal tissue, bile ducts, and hepatocytes, and is generally regarded as a developmental anomaly of the bile duct plate and surrounding mesenchyme [1, 2]. MHL predominantly affects infants and young children and represents a substantial proportion of benign pediatric liver tumors [1]. In contrast, adult-onset MHL is exceedingly rare, with only a small number of cases reported in the literature [3–6].

Adult MHL presents unique diagnostic challenges because its clinical and radiological features often differ from those of pediatric cases. Whereas childhood MHL typically manifests as a large multicystic mass, adult lesions may be predominantly solid and can closely mimic a broad spectrum of hepatic neoplasms, including hepatocellular tumors (e.g. hepatocellular adenoma, focal nodular hyperplasia, or hepatocellular carcinoma), biliary epithelial/cholangiocytic tumors, and mesenchymal tumors [3–6]. This overlap frequently leads to difficulty in preoperative diagnosis, particularly in young women, in whom a history of oral contraceptive use may further bias the radiological interpretation toward benign hepatocellular neoplasms. In addition, adult MHL may show progressive or rapid enlargement, raising concern for malignant transformation despite its benign nature. Given the diagnostic uncertainties and the risks of malignant potential and tumor growth, surgical resection is a reasonable option.

Herein, we report a rare case of adult MHL in a 31-year-old woman with a history of oral contraceptive use, presenting as a rapidly enlarging, predominantly solid liver tumor initially suspected to be a hepatic adenoma.

## Case report

A 31-year-old woman with no history of liver disease or significant comorbidities was referred to our institution for evaluation of a liver tumor. She was a non-smoker and consumed alcohol only occasionally. Her past medical history was notable for dysmenorrhea, for which she had been taking a low-dose combined oral contraceptive pill for more than 10 years. There was no family history of liver disease or malignancy. In 2024, screening abdominal ultrasonography as a medical check-up revealed a 23-mm hyperechoic lesion in segment 7 of the liver (Fig. 1).

**Figure 1:**
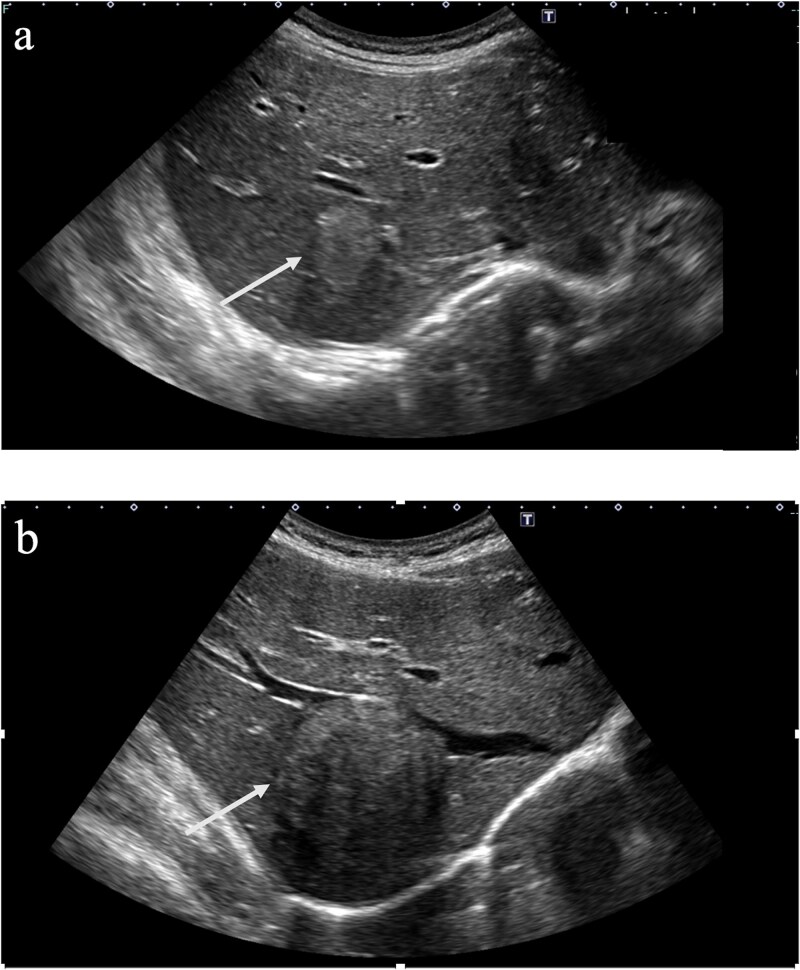
Abdominal ultrasonography revealed a 23-mm hyperechoic mass (a, arrow) in hepatic segment 7, which enlarged to 68 mm (b, arrow) 1 year later.

Further evaluation was recommended, but she did not undergo additional medical evaluation at that time. One year later, abdominal ultrasonography showed that the same lesion had enlarged to 68 mm in diameter, and she was referred to the Department of Gastroenterology at our hospital.

On examination, she was asymptomatic, with no abdominal pain, palpable mass, or constitutional symptoms. Physical examination was unremarkable, and there were no signs of chronic liver disease. Laboratory tests, including liver function tests, coagulation profile, and viral hepatitis serologies, were within normal limits. Serum α-fetoprotein, protein induced by vitamin K absence or antagonist-II, carcinoembryonic antigen, and carbohydrate antigen 19-9 were all within reference ranges. Tumor biopsy was not performed because of the risk of dissemination. Contrast-enhanced computed tomography (CT) demonstrated a well-circumscribed tumor with heterogeneous internal enhancement that gradually increased from the arterial phase to the delayed phase. On Gadoxetic acid-enhanced magnetic resonance imaging (EOB–MRI), the lesion showed reduced gadoxetic acid uptake in the hepatobiliary phase, no restricted diffusion on diffusion-weighted imaging, and heterogeneously high signal intensity on T2-weighted imaging (Fig. 2).

**Figure 2:**
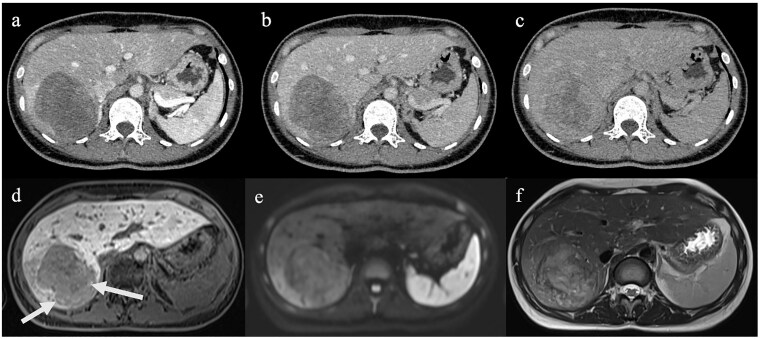
A 76-mm mass was identified in hepatic segment 7 on CT (a–c) and MRI (d–f). Contrast-enhanced CT demonstrated a mass with heterogeneous internal enhancement that gradually increased from the arterial phase to the delayed phase. (a) Arterial phase (b) portal venous phase (c) delayed phase EOB-MRI revealed a 76-mm mass with predominantly reduced uptake in the hepatobiliary phase and focal hyperintense areas suggestive of gadoxetic acid uptake (arrow). The lesion was isointense on DWI and showed heterogeneous hyperintensity on T2-weighted imaging. (d) Hepatobiliary phase on EOB-MRI (e) DWI (f) T2-weighted imaging. DWI, diffusion-weighted imaging.

Given the rapid growth and proximity to the right hepatic vein, the patient underwent robot-assisted extended posterior sectionectomy with en bloc right hepatic vein resection. The postoperative course was uneventful. Histopathological examination revealed a MHL, characterized by fibromyxoid stroma containing spindle-shaped mesenchymal cells and bile duct–like structures, with scattered entrapped hepatocyte plates and numerous blood vessels (Fig. 3).

**Figure 3:**
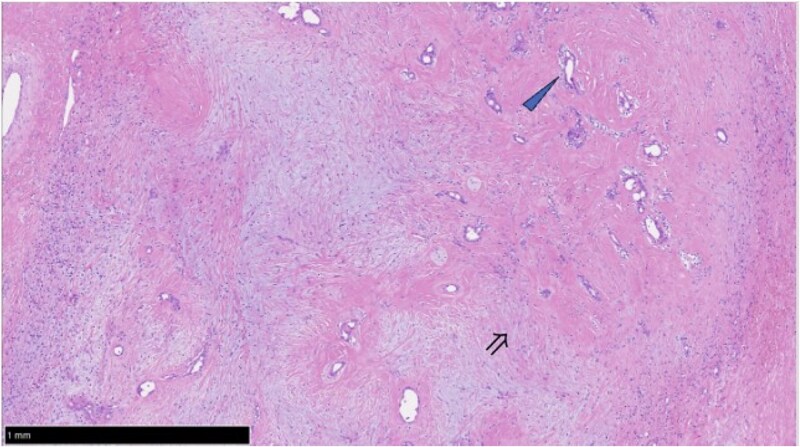
Histopathological findings on hematoxylin and eosin staining. Hematoxylin and eosin staining revealed abundant fibromyxoid to fibrous stroma containing scattered irregular bile duct–like structures (arrowhead) and spindle-shaped mesenchymal cells (double arrow). No significant cytologic atypia, necrosis, or increased mitotic activity was identified.

## Discussion

Adult MHL is exceedingly rare and may be discovered incidentally or present with non-specific symptoms such as abdominal pain [3–8]. In our patient, the lesion was detected on screening ultrasonography and demonstrated rapid enlargement over 1 year, which heightened concern for malignancy despite the lack of overt radiologic features of aggressive disease.

Preoperative diagnosis is challenging because imaging findings are heterogeneous and overlap with multiple entities. In particular, adult MHL can be mistaken for benign cystic lesions (most commonly hemorrhagic hepatic cysts) as well as cystic biliary neoplasms such as mucinous cystic neoplasm, hydatid disease, and cystic metastases [2, 5, 9]. Adult tumors are more variable and may be mixed solid–cystic or predominantly solid, closely mimicking hepatocellular adenoma, focal nodular hyperplasia, hemangioma, or metastatic tumors [3–7, 10]. In our patient, dynamic CT and MRI showed a well-circumscribed predominantly solid lesion with gradual heterogeneous enhancement—findings that were not sufficiently specific to establish a confident preoperative diagnosis.

EOB-MRI is useful for characterizing focal liver lesions because the hepatobiliary phase reflects hepatocyte function and transporter activity [11]. Hepatobiliary-phase hyperintensity typically favors focal nodular hyperplasia and some well-differentiated hepatocellular neoplasms, whereas most non-hepatocellular lesions appear hypointense [11]. MHL is reported to be predominantly hypointense on the hepatobiliary phase [9]. In our case, the lesion showed overall reduced gadoxetic acid uptake in the hepatobiliary phase, with faint peripheral uptake. Considering the patient’s young age, long-term oral contraceptive use, and the suggestion of a hepatocellular component on MRI (including hepatobiliary-phase behavior and possible fat), a hepatocellular lesion remained a leading consideration; however, the imaging pattern was not typical for a classic hepatocellular adenoma, and an “unclassified” hepatocellular adenoma versus other benign hepatocellular tumors was considered. This diagnostic uncertainty illustrates that adult MHL can mimic benign hepatocellular tumors in young women and requires careful correlation with growth kinetics and ancillary imaging features.

Rapid enlargement further complicated management. The tumor increased from 2 cm to more than 7 cm within 1 year, raising concern for malignant transformation [2, 3, 5, 6, 12]. Although MHL is considered benign, a pathogenetic link with undifferentiated embryonal sarcoma has been proposed, and malignant transformation has been reported, predominantly in pediatric cases [8, 12, 13]. Importantly, rapid growth without malignant transformation has also been described in adult MHL, and tumor enlargement alone should not be regarded as definitive evidence of malignancy [3–6].

Surgical resection is generally recommended for MHL because of mass effect and the debated but possible malignant potential [2, 6–8, 10, 12–14]. Shared glypican-3 expression in undifferentiated embryonal sarcoma and MHL has also been reported [15]. Outcomes after R0 resection are typically favorable, although recurrence has been reported [7, 8]. In the present case, rapid growth and proximity to the right hepatic vein supported upfront resection. Robot-assisted anatomical extended posterior sectionectomy with en bloc right hepatic vein resection enabled complete removal and an uneventful recovery. Minimally invasive approaches may be advantageous for posterior segment lesions abutting major hepatic veins by facilitating precise dissection and secure vascular control while preserving functional parenchyma.

In summary, adult MHL should be considered in young adults with a well-circumscribed liver mass when a benign hepatocellular tumor is highly suspected, even when hepatobiliary-phase findings are not supportive of hepatocellular lesions.
